# Impact of Global Budget Revenue Policy on Emergency Department Efficiency in the State of Maryland

**DOI:** 10.5811/westjem.2019.8.43201

**Published:** 2019-10-14

**Authors:** Ai Ren, Bruce Golden, Frank Alt, Edward Wasil, Margret Bjarnadottir, Jon Mark Hirshon, Laura Pimentel

**Affiliations:** * University of Maryland, Robert H. Smith School of Business, Decision, Operations, and Information Technologies, College Park, Maryland; † American University, Kogod School of Business, Department of Information Technology and Analytics, Washington, District of Colombia; ‡ University of Maryland School of Medicine, Department of Emergency Medicine, Baltimore, Maryland

## Abstract

**Introduction:**

On January 1, 2014, the State of Maryland implemented the Global Budget Revenue (GBR) program. We investigate the impact of GBR on length of stay (LOS) for inpatients in emergency departments (ED) in Maryland.

**Methods:**

We used the Hospital Compare data reports from the Centers for Medicare and Medicaid Services (CMS) and CMS Cost Reports Hospital Form 2552-10 from January 1, 2012–March 31, 2016, with GBR hospitals from Maryland and hospitals from West Virginia (WV), Delaware (DE), and Rhode Island (RI). We implemented difference-in-differences analysis and investigated the impact of GBR implementation on the LOS or ED1b scores of Maryland hospitals using a mixed-effects model with a state-level fixed effect, a hospital-level random effect, and state-level heterogeneity.

**Results:**

The GBR impact estimator was 9.47 (95% confidence interval [CI], 7.06 to 11.87, p-value<0.001) for Maryland GBR hospitals, which implies, on average, that GBR implementation added 9.47 minutes per year to the time that hospital inpatients spent in the ED in the first two years after GBR implementation. The effect of the total number of hospital beds was 0.21 (95% CI, 0.089 to 0.330, p-value = 0 .001), which suggests that the bigger the hospital, the longer the ED1b score. The state-level fixed effects for WV were −106.96 (95% CI, −175.06 to −38.86, p-value = 0.002), for DE it was 6.51 (95% CI, −8.80 to 21.82, p-value=0.405), and for RI it was −54.48 (95% CI, −82.85 to −26.10, p-value<0.001).

**Conclusion:**

Our results indicate that GBR implementation has had a statistically significant negative impact on the efficiency measure ED1b of Maryland hospital EDs from January 2014 to April 2016. We also found that the significant state-level fixed effect implies that the same inpatient might experience different ED processing times in each of the four states that we studied.

## INTRODUCTION

The escalating cost of healthcare in the United States is unsustainable. In 2016 spending reached 17.9% of the gross domestic product, or $10,348 per person. 1 Many studies on healthcare reform in the U.S. focus on the factors driving the nation’s high level of expenditure. 2 – 6 The payment system is the subject of one major stream of research.

### All-Payers Payment System and Total Patient Revenue

The State of Maryland is at the forefront of healthcare reform in the U.S. The state is unique in its implementation of an all-payers payment system for hospitals. The system is governed by the Health Services Cost Review Commission (HSCRC), which sets hospital rates for all providers for both inpatient and outpatient services. 7 In 1977 the federal government granted the state a Medicare waiver that required government payers to abide by HSCRC hospital rates. Global Budget Revenue (GBR) is a revision of this waiver and was implemented in 2014. GBR drives a value-based healthcare service by setting global budgets for acute care hospitals, i.e., creating a capitated system for hospitals.

In 2011, Maryland implemented the Total Patient Revenue (TPR) program, a revenue constraint policy designed by the HSCRC. TPR was implemented as a pilot project in 12 Maryland hospitals located primarily in rural and geographically isolated parts of the state. Under TPR, these pilot hospitals were guaranteed a certain annual revenue calculated from a formula based on the prior year’s revenue and reasonable annual adjustments. This structure provided an incentive to control costs by reducing unnecessary hospitalizations and inpatient resources. Communities were rewarded for the development of robust outpatient resources and improving the health of the population. Based on the success of TPR, the state and federal government moved forward with GBR on a statewide basis.

### Global Budget Revenue

On January 1, 2014, the State of Maryland began the GBR program with the main goals of improving the health of communities, improving the patient experience, and lowering the cost of healthcare services for all patients. In contrast to the 36-year-old waiver policy that preceded it, GBR guarantees a hospital’s annual revenue by calculating global budget based on market share. Adjustments in global budgets are tied to changes in market share and the state’s gross domestic product. In some ways, GBR is an extension of TPR. However, GBR is not a voluntary program; it requires every Maryland hospital to participate. The main difference is that TPR was implemented in geographically isolated areas of the state where catchment areas are clear. Hospitals under GBR operate in more competitive market environments. 7 In the online appendix, Table A1 lists the names of the Maryland hospitals that are under the GBR program.

In the past, hospital revenue was directly linked to the number of medical services that the hospital provided. In contrast, under GBR and TPR, each hospital’s total annual revenue is defined by the HSCRC and known at the beginning of each fiscal year. The hospital margin is the difference between the global budget and annual cost. As a result, hospitals are motivated to control costs while maintaining or growing market share. 7 – 10

### Medicaid Expansion

Medicaid is a state and federal jointly-funded healthcare insurance program for low income Americans. The Medicaid program was expanded to individuals with annual incomes below 138% of the federal poverty level when the Patient Protection and Affordable Care Act (also referred to as the Affordable Care Act [ACA] or Obamacare) was passed. Maryland is one of 33 states that adopted the Medicaid expansion. This ACA provision was implemented on January 1, 2014, 10 days before GBR began.

**Population Health Research Capsule**

What do we already know about this issue?

*In January 2014 Maryland began the Global Budget Revenue (GBR) program. Its goals include improving the health of communities and patient experience while lowering costs*.

What was the research question?

What was the impact of GBR on emergency department performance and efficiency in Maryland?

What was the major finding of the study?

*At the patient level, GBR implementation correlates with longer ED length of stay for admitted patients*.

How does this improve population health?

*Our results indicated that GBR implementation had a statistically significant negative impact on the efficiency performance of Maryland hospital EDs*.

### Emergency Department Efficiency

Emergency departments (ED) have taken on an increasingly important role in the healthcare system and are often cited as a key contributor to rising costs. 8, 11 The ED is an important hospital-based service; GBR, because of its focus on cost control, could have an impact on ED efficiency. We selected the length of stay (LOS) or ED1b (efficiency measure); see online appendix section A3) for admitted patients as our dependent variable. LOS is a Centers for Medicare and Medicaid Services (CMS) metric designed to measure the impact of hospital throughput on ED patients. Multiple studies document the deleterious effect of prolonged ED stays on quality of care. 12 – 14

Our research focused on the impact of GBR on ED performance and efficiency in Maryland. Our study was confounded by the nearly simultaneous implementation of Medicaid expansion with GBR. To control for the effect of Medicaid expansion on Maryland’s EDs, we compared our results with three geographically proximate states that had also adopted Medicaid expansion: West Virginia (WV), Delaware (DE), and Rhode Island (RI).

## METHODS

### Data

GBR was implemented on January 1, 2014. Our study period ran from January 1, 2012–March 31, 2016. We define January 1, 2012–September 30, 2013, as the pre-treatment period and April 1, 2014–March 31, 2016, as the post-treatment period. The six-month gap between September 2013 and April 2014 is omitted from our study and represents the transition period of GBR implementation and Medicaid expansion. As we used publicly available, administrative datasets that do not include data about individuals, institutional review board review was not sought.

### Data Sources

The overall data for our study combines three datasets. The first dataset uses data from the CMS Cost Reports Hospital Form 2552-10. 15 This form is generated by Medicare-certified institutional providers and is required in order to achieve settlement of costs (in total and for Medicare). 15 From the variables available in these reports, we chose TOTAL_HOSPITAL_BEDS, which is the total number of hospital beds during the fiscal year.

The second dataset is the CMS Hospital Compare data. 16 This dataset has a variety of reports about the quality of care delivered by hospitals. We used two of these reports: Emergency Department Throughput before July 17, 2014, and Timely and Effective Care after July 2014. These reports contain many measurement scores including ED1b. We used the hospital-level ED1b score as our main outcome variable and state-level annual reports to describe the trend among the four states studied. Table 1 presents the CMS Hospital Compare data reports that we used and their measurement periods.

GBR agreements were signed on July 1, 2013, and hospitals were able to extend the implementation deadline until October to make modifications. This means that hospitals could have implemented GBR at different times, so we designated a six-month window (October 1, 2013–March 31, 2014) as the treatment implementation period.

The third dataset is the Kaiser Family Foundation (KFF) database. 17 KFF is a non-profit organization focusing on national health issues that provides data for policy analysis and research. The data and reports that we used were the following: Hospital Beds per Thousand Population 2012–2015, 18 – 20 Hospital Emergency Room Visits per Thousand Population 2012–2015, 21 and Total Medicaid Managed Care Enrollment 2011–2015. 22 These data and reports all provide state-level information.

Merging the data gave us a total of 353 ED1b reports from Maryland, WV, RI, and DE from January 1, 2012–March 31, 2016. There are 24 reports from DE, 135 from Maryland, 44 from RI, and 150 from WV.

### Methodology

In this study, we used the difference-in-differences method (DID), which is widely used in healthcare management and policy analysis. 23 – 28 DID determines two differences and calculates the treatment or policy effect by determining the difference of the two differences. Examples of studies using DID include that work by Tiemann and Schreyogg on the impact of privatization on hospital efficiency in Germany. 27 Buchner et al. used DID to study the impact of health system entry on hospital efficiency and profitability. 28

In our study, the first difference is the comparison of a GBR, hospital’s performance before and after GBR implementation. The second difference is the comparison of scores from a group of control hospitals in the same time frame. Finally, we used the second difference from the control group to rule out the part of the first score difference that is not influenced by GBR. This allowed us to estimate the treatment effect within the treatment group. More precisely, GBR adoption was considered the treatment, the hospitals implementing GBR constituted the treatment group, and hospitals not implementing GBR but otherwise similar (in their adoption of Medicaid Expansion, for example) were considered the control group. This allowed us to identify the treatment effect due to the impact of GBR as opposed to Medicaid expansion or other industry-wide trends.

The treatment group was all Maryland hospitals that adopted GBR on January 1, 2014, but did not participate in the TPR program. According to the Annual Report on Selected Maryland General and Special Hospital Services Fiscal Year 2016, 10 Maryland has 46 EDs located in general hospitals. Of those 46 hospitals, 10 rural hospitals have participated in the TPR program since July 2010 and are, therefore, excluded from the analysis. The control group includes hospitals from WV, RI, and DE. These three states adopted the original Medicaid expansion on January 1, 2014, at the same time as Maryland, but did not implement the GBR or TPR programs. The main reason that we chose these three as our control group is that Medicaid expansion might have caused and been accompanied by some unmeasurable changes in patient behavior. For example, people who were newly eligible for Medicaid after the expansion would have had different strategies for choosing healthcare providers. We assumed that people from the four states exhibited similar patterns in their reactions to Medicaid expansion. The online appendix section A2 provides the logic behind the selection of the control group.

### Model and Setup

We formatted the final dataset into an unbalanced panel dataset and implemented a mixed-effects, linear regression model with a state-level fixed effect, a hospital-level random effect, and state-level heterogeneity to investigate the impact of GBR implementation on the ED1b scores of Maryland hospitals. The variables considered in our model are listed in Table 2 (see online appendix sections A4 and A5 for more details on our model).

### Sensitivity Analysis

We conducted three types of sensitivity analysis. The first analysis assessed whether our treatment effect estimates were sensitive to the length of the first report period. In our study, we used four CMS Hospital Compare data reports. The first report covers the nine-month period from January 1, 2012–September 30, 2012, and the remaining three are 12-month period reports. Then, we introduced the report length into the model for the sensitivity analysis.

Second, to assess whether our estimates were sensitive to each state in the control group, we conducted three sensitivity analyses using three alternative control groups. In the first alternative control group, we removed the hospitals in WV counties with smaller populations (less than 45,000), since Maryland counties in our study have at least 45,000 residents. This left 18 WV hospitals in the control group. In the other two alternative control groups, we removed all hospitals from RI and then from DE. Third, we conducted a robustness check on the relationship between the number of hospital beds and resulting ED1b score by dropping hospitals with more than 500 beds.

## RESULTS

### Model Results

Table 3 summarizes some general information about the four states involved in our study.

Table 4 shows the coefficient estimates under the panel data setting with mixed effects, clustered error on the state level using generalized least squares (GLS). The estimated coefficient of the variable *tt* (GBR impact estimator) was 9.466872 with a *p*-value<0.001, implying that GBR had a statistically significant impact on ED LOS (ED1b) in Maryland’s GBR hospitals. On average, GBR implementation added 9.47 minutes per year to the time hospital inpatients spend in the ED after controlling other factors in the model. As shown in the results, WV (*wv coef. =* −106.9579, *p*-value = 0.002) had the best overall ED1b performance, and RI (*ri coef*.=−54.47875, *p*-value <0.001) performed the second best among the four states.

**Figure 1:**
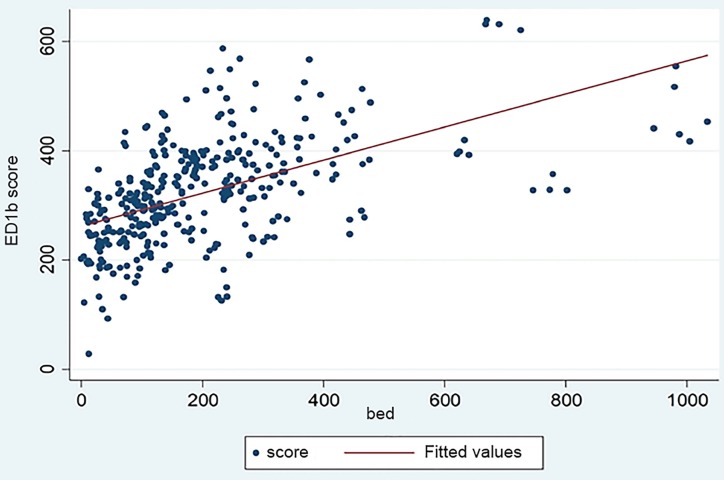
Scatter plot of ED1b scores vs total number of hospital beds.

The significant *p*-values of variables *wv* and *ri*, which are state-level, fixed-effect variables, imply that patients admitted through EDs with similar medical problems and conditions from WV, RI, and Maryland might experience significantly different time in EDs. The effect of the total number of hospital beds (*bed coef. =* 0.2096206, *p*-value = 0.001), which is positively associated with ED1b scores as shown in Figure 1, provides strong evidence that the bigger the hospital, the longer the ED1b. The time variable (*t coef*. = 1.393201, *p*-value <0.001) implies that during our study period, the hospitals’ ED1b performance, on average, became worse overall in all four states.

### Sensitivity Analyses

In the first sensitivity analysis, we introduced the report length into the model. The regression estimator for length (*length coef*. = −1.341053, *p*-value = 0.384) implies the shorter time period in the first report does not impact the ED1b score. (See online appendix, Table A9.) In the second sensitivity analysis, we introduced the total number of registered nurses per thousand population and the hospital beds per thousand population at the state level to describe the changes in available healthcare resources. Table 5 summarizes the sensitivity analysis estimates for the GBR effect. The regression results show that adding the two new variables or using the three alternative control groups is consistent with our main results. The incremental time estimate for each model in Table 5 is approximately nine minutes.

Fourth, after dropping hospitals with more than 500 beds, the number of hospital beds (*bed coef*. = 0.270943, *p*-value<0.001) is still positively associated with a hospital’s ED1b score. Online appendix section A6 provides the details of the sensitivity analysis.

## DISCUSSION

At the patient level, GBR implementation correlates with longer ED LOS for patients being admitted to the hospital. We believe that this implies that GBR has fundamentally changed the way emergency physicians and hospital staff approach the hospitalization decision. The Evaluation of the Maryland All-Payer Model Second Annual Report funded by CMS in 2017 emphasized that GBR targeted both healthcare cost and quality. 9 The model has encouraged more workup and interface with case managers in the ED; the objective is to ensure patient safety and high-quality care in the community in lieu of admission for appropriate patients. These changes were likely contributing factors to the increase in the total timespan for the care of an ED patient. Future work includes a study on whether and how Maryland hospital EDs adopted new strategies or modified their procedures for healthcare service delivery in response to the implementation of GBR. It remains to be seen if the changes in Maryland hospital EDs had or will have a substantial impact on Maryland’s healthcare system.

We found significant differences among the three Medicaid expansion states to which Maryland was compared. WV and RI had significantly shorter ED1b scores for admitted patients than Maryland. Delaware’s score was slightly longer. After applying sensitivity analysis using three alternative control groups, we found that the difference between Maryland’s ED1b and those different control groups remained significant. GBR, a state policy, is correlated with longer LOS for admitted patients. In our study, the state-level fixed effect is significant. Nevertheless, there may well be unidentified confounders that influenced our results.

According to Benjamin C. Sun, professor of emergency medicine at Oregon Health and Science University in Portland, “It’s not really fair to compare, say, a public teaching hospital in the middle of New York City that sees 120,000 patients with one that is in a rural area that sees 5,000 patients.” 29 Similarly, it may not be fair to simply compare ED scores across states. Our comparison across states assumes similar demographics and disease burdens, both of which could affect hospital utilization. Also, we are assuming similar admission practices across states. More particularly, we assume the changes in Maryland inpatient census other than affected by the implementation of Medicaid expansion and GBR can be controlled by our control group. In February 2017, a news report stated that “Maryland ER wait times are the worst in the nation,” a conclusion derived by simply comparing the ED scores published by CMS Hospital Compare. 30 Viewed in this light, interpreting the significant state-level fixed effect obtained in our study without clarifying factors that may be unique or particular to each state, might confuse, rather than clarify, perceptions of hospital ED performance.

## LIMITATIONS

We acknowledge several other limitations in our study. The GBR policy was adopted on January 1, 2014, 10 days after Maryland began Medicaid expansion. The control group hospitals then had to come from neighboring states that also implemented traditional Medicaid expansion at the same time, thus, limiting our control group to WV, RI, and DE. Of these states, RI and DE have few hospitals. Another limitation was the incomplete report data. Overall, the reporting rate of the control group is 75%. According to KFF Total Hospital Reports, 31 there should be 290 Hospital Compare data reports from CMS. However, we found only 218 complete reports. It is possible that the missing data might have some impact on our results.

Another limitation is the possibility that unmeasured confounding factors may have affected ED LOS. Factors such as hospital closures, demographics, or shifts in access to care could have affected our results. To eliminate the effect of those possible confounding factors, the ideal measure would be the volume of each hospital’s ED visits. CMS started to collect volume data on January 22, 2015. However, some states in our study only started to report this measure on November 10, 2016. Therefore, we selected features other than volume data and note that we might not have been able to eliminate all effects.

We were also limited in our choice of performance measure ED1b, which reflects the total time inpatients spend in the ED. Ideally our study would examine both ED1b and the corresponding outpatient measure, OP18. However, CMS only maintains Maryland State OP18 reports going back to January 1, 2014. As there is no data for the pre-treatment period, we cannot study the impact of GBR on the OP18 measure. Our design assumed that residents living in the four geographically close states shared similar reaction patterns to the Medicaid expansion. Then, from an aggregate point of view at the hospital level, we assumed that our control group could rule out the impact of Medicaid expansion on Maryland ED LOS. It is possible that not every hospital was affected by Medicaid expansion at the same proportion, which might have affected the estimates. Also, our secondary finding, the significant difference in time spent in EDs across the four states, should be further investigated by analyzing data from the Nationwide Emergency Department Database.

## CONCLUSION

We conducted an empirical analysis of the impact of GBR implementation on Maryland ED efficiency measure ED1b from January 2014–April 2016. Our results indicated that GBR implementation had a statistically significant negative impact on the efficiency performance of Maryland hospital EDs. The mean 2014 ED1b score was 398.6 minutes, and our study showed an average increase of 2.4%, or 9.47 minutes per year, in the first two years after the implementation of GBR. We also found that the significant state-level fixed effect implies that the same inpatient might experience different ED processing times in each of the four states that we studied. Further research is indicated to explore the dynamics of GBR including the reasons for increasing ED length of stay.

## Supplementary Information



## Figures and Tables

**Table 1:** CMS Hospital Compare data report dates and measurement periods.

Report ID	Measurement period
20130701	1/1/2012–9/30/2012 (pre)
20140717	10/1/2012–9/30/2013(pre)
20151210	4/1/2014–3/31/2015 (post)
20161219	4/1/2015–3/31/2016 (post)

**Table 2:** Variables considered in our model.

Variables	Description
*ED1b*	Hospital’s ED1b score
*ri*	Indicator variable for Rhode Island
*wv*	Indicator variable for West Virginia
*de*	Indicator variable for Delaware
*bed*	Number of beds in the hospital
*t*	Time
*period*	Indicator variable for post-treatment period
*tt*	GBR impact estimator
*medicaid*	Medicaid enrollment percentage of the population in each state
*edvperpop*	Hospital emergency department visits per thousand population of each state
*const*	Constant

**Table 3:** Hospitals per 10,000 population.

State	Number of hospitals*	Population (2013)**	Hospitals per 10,000 population
Maryland	50	5,928,814	0.084
West Virgina	54	1,854,304	0.29
Rhode Island	11	1,051,511	0.10
Delaware	7	925,749	0.076

* 1999–2015 American Hospital Association Survey.^19^

** Annual estimates of the resident population for states 2013.^18^

**Table 4:** Regression results from panel setting with mixed effects and state-level heterogeneity (GLS estimator).

Variables	Coefficient Estimator	Confidence interval (95%)
*ri*	−54.47875**	(−82.85274, −26.10476)
*wv*	−106.9579*	(−175.0596, −38.85614)
*de*	6.510064	(−8.798204, 21.81833)
*t*	1.393201**	(0.721995, 2.064408)
*tt*	9.466872**	(7.062948, 11.8708)
*bed*	0.2096206**	(0.0893118, 0.3299294)
*medicaid*	−0.6514587	(−1.663111, 0.3601937)
*edvperpop*	−0.0064578	(−0.2680708, 0.2551552)
*const*	342.5963**	(239.5974, 445.5952)

* p-value ≤0.01,

** p-value ≤0.001

*GLS*, generalized least squares.

**Table 5:** Summary of the sensitivity analysis.

Model	GBR Effect Estimate	95% Confidence Interval
Mixed model	9.466872*	(7.062948, 11.8708)
Adding length of report period	8.480697*	(7.851343, 9.110051)
Adding registered nurses per thousand population and hospital beds per thousand population	8.825362*	(7.197024, 10.4537)
Control group with partial WV	10.90678*	(6.785388, 15.02817)
Control group without DE	9.85151*	(7.934328, 11.76869)
Control group without RI	8.422524*	(7.44502, 9.400028)

* p-value ≤0.001

*GBR*, Global Budget Revenue; *WV*, West Virginia; *DE*, Delaware; *RI*, Rhode Island.
