# Supplementary material for: Impact of Global Budget Revenue Policy on Emergency Department Efficiency in the State of Maryland
Source: West J Emerg Med. 2019 Oct 14;20(6):885–92. doi: 10.5811/westjem.2019.8.43201 (PMC6860385; doi:10.5811/westjem.2019.8.43201)
Supplement: Supplementary file 1 [file wjem-20-885-s001.docx]

**A1. DETAILS OF THE GLOBAL BUDGET REVENUE PROGRAM**

**A1.1 List of GBR hospitals**

Table A1 lists the names of the Maryland hospitals that are under the Global Budget Revenue policy as well as the county, county population in 2012,^1^ number of hospital beds (fiscal year 2014-2015^2^), and employees on payroll (fiscal year 2014-2015^2^).

**Table A1.** Maryland Hospitals under GBR.

| Hospital Name | County | County Population (2012) | Number of Beds | Employees on Payroll |
| --- | --- | --- | --- | --- |
| Anne Arundel Medical Center | ANNE ARUNDEL | 550175 | 384 | 2651.95 |
| Atlantic General Hospital | WORCESTER | 51567 | 48 | 664.3 |
| Bon Secours Hospital | BALTIMORE CITY | 622417 | 88 | 683.75 |
| Doctors’ Community Hospital | PRINCE GEORGES | 881419 | 182 | 1139.85 |
| Fort Washington Hospital | PRINCE GEORGES | 881419 | 31 | 261.37 |
| Frederick Memorial Hospital | FREDERICK | 239520 | 258 | 1490.83 |
| Greater Baltimore Medical Center | BALTIMORE | 817682 | 287 | 2272 |
| Holy Cross Hospital | MONTGOMERY | 1004476 | 440 | 2615.59 |
| Holy Cross Germantown Hospital | MONTGOMERY | 1004476 | 93 | 486.16 |
| Howard County General Hospital | HOWARD | 248540 | 259 | 1433.9 |
| Johns Hopkins Bayview Medical Center | BALTIMORE CITY | 622417 | 442 | 2646.1 |
| Johns Hopkins Hospital | BALTIMORE CITY | 622417 | 993 | 9333.94 |
| Laurel Regional Hospital | PRINCE GEORGES | 881419 | 124 | 624.9 |
| MedStar Franklin Square Medical Center | BALTIMORE | 817682 | 376 | 2851 |
| MedStar Good Samaritan Hospital | BALTIMORE CITY | 622417 | 287 | 1505 |
| MedStar Harbor Hospital | BALTIMORE CITY | 622417 | 120 | 1042 |
| MedStar Montgomery Medical Center | MONTGOMERY | 1004476 | 120 | 820 |
| MedStar Southern Maryland Hospital Center | PRINCE GEORGES | 881419 | 207 | 1193.28 |
| MedStar St. Mary’s Hospital | SAINT MARYS | 108993 | 82 | 896 |
| MedStar Union Memorial Medical Center | BALTIMORE CITY | 622417 | 205 | 1999 |
| Mercy Medical Center | BALTIMORE CITY | 622417 | 233 | 2594.5 |
| Northwest Hospital Center | BALTIMORE | 817682 | 245 | 1189.53 |
| Peninsula Regional Medical Center | WICOMICO | 100521 | 275 | 2325.7 |
| Prince George County Hospital | PRINCE GEORGES | 881419 | 225 | 1564.28 |
| Shady Grove Adventist Hospital | MONTGOMERY | 1004476 | 340 | 1658.04 |
| Sinai Hospital of Baltimore | BALTIMORE CITY | 622417 | 428 | 3537.29 |
| St. Agnes Hospitals | BALTIMORE CITY | 622417 | 288 | 2455.2 |
| Suburban Hospital | MONTGOMERY | 1004476 | 220 | 1353.75 |
| University of Maryland Baltimore Washington Medical Center | ANNE ARUNDEL | 550175 | 310 | 2147.3 |
| University of Maryland Charles Regional Medical Center | CHARLES | 150710 | 115 | 708.47 |
| University of Maryland Harford Medical Center | HARFORD | 248540 | 89 | 616 |
| University of Maryland Medical Center | BALTIMORE CITY | 622417 | 715 | 6995.28 |
| University of Maryland Medical Center Midtown | BALTIMORE | 622417 | 190 | 1107.79 |
| University of Maryland St. Joseph Medical Center | BALTIMORE | 622417 | 258 | 1556.26 |
| University of Maryland Upper Chesapeake Medical Center | HARFORD | 248540 | 181 | 1415 |
| Washington Adventist Hospital | MONTGOMERY | 1004476 | 242 | 1092.98 |

**A1.2 Hospital payment**

Under the Maryland all-payer model, the Heath Services Cost Review Commission (HSCRC) provides each hospital with an approved annual global budget at the beginning of the fiscal year. The hospital-approved regulated revenue is calculated from the Base Year (which is the year or years before the agreement effective year) and adjustments. The adjustments compensate for several items such as annual cost increases, update factors, performance-based rewards or penalties, population, and demographic change.^3^ Each hospital is expected to meet its specified revenue amount, and penalties are applied when the hospital is over or under its approved budget.

A theoretical example of a hospital exceeding its approved budget is shown in Figure A1.^3^ Assuming an agreement signed between Hospital X and HSCRC on July 14, 2014 provided Hospital X with an approved total revenue $1,636,470,794 and the actual revenue was $1,693,747,271. Then, the overage amount will be $1,693,747,271-$1,636,470,794=$57,276,477 and the overage rate will be $57,276,477/$1,636,470,794≈3.5%. According to the penalty formula, in addition to penalizing the overage, there is an additional penalty of 20% to be applied to the overage that falls between 0.5% and 1% of the approved revenue. Similarly, a penalty of 50% will be applied to the overage between 1% and 2%, and 100% penalty will be applied to any overage beyond 2%. Since overage in this example is 3.5%, the penalty will be $1,636,470,794×(1%-0.5%)×20%+$1,636,470,794×(2%-1%)×50%+($57,276,477-$1,636,470,794×2%)×100%=$34,365,886. Adding the overage amount ($57,276,477), a $91,642,363 deduction would be applied to Hospital X’s approved revenue for the next year.


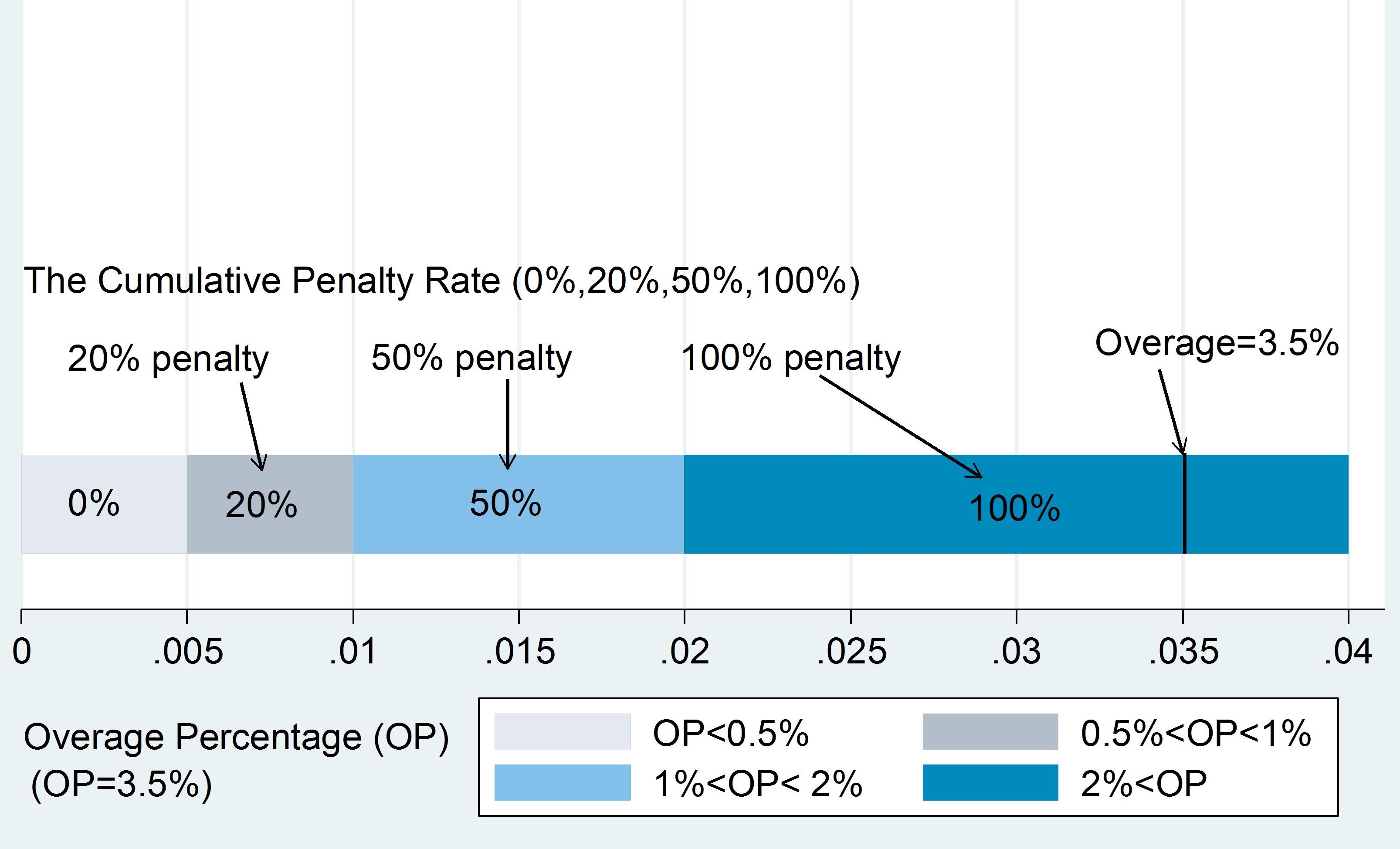


**Figure A1.** Example of a Hospital Exceeding Approved Revenue by 3.5%.

**A2. MATCHING OF MARYLAND AND CONTROL STATES**

**A2.1 Identifying states that implemented Medicaid expansion on January 1, 2014**

Table A2 lists the 12 states that are located along the central east coast and relatively close to Maryland.^4,5^ It also lists whether the state or district implemented Medicaid expansion and if so, when.

**Table A2.** Central East Coast States and Districts – Medicaid Expansion.

| Abbreviation | State Name | Medicaid Expansion | Date |
| --- | --- | --- | --- |
| CT | Connecticut | Yes | 4/1/2010 |
| DE | Delaware | Yes | 4/1/2014 |
| DC | District of Columbia | Yes | 7/1/2010 |
| MD | Maryland | Yes | 4/1/2014 |
| MA | Massachusetts | Yes | 4/1/2014 |
| NJ | New Jersey | Yes | 4/14/2011 |
| NC | North Carolina | No | N/A |
| PA | Pennsylvania | Yes | 2/1/2015 |
| RI | Rhode Island | Yes | 4/1/2014 |
| VT | Vermont | Yes | 4/1/2014 |
| VA | Virginia | Yes | 5/30/2018 |
| WV | West Virginia | Yes | 4/1/2014 |

**A2.2 Choosing control states**

Our study is complicated by the fact that Maryland began Medicaid expansion on January 1, 2014. According to a recent review, there are more than 200 papers studying the impact of Medicaid expansion throughout the US. Of these papers, some of the more recent are extensions of earlier Medicaid expansion studies that were revised by adding new data and continue to support earlier findings.^6^ One Maryland-based study found no significant relationship between Medicaid expansion and changes in total Emergency Department (ED) volume by hospital.^7^ Another study in Illinois found an increase in total ED visits after the Affordable Care Act (ACA) implementation.^8^ Since the overall conclusions vary from state to state, we believe Medicaid expansion might have different impacts on different states in terms of ED visit volume because residents of different states might have different reactions to changes in healthcare policy. In particular, people who were newly eligible for Medicaid after the expansion may have different strategies for choosing healthcare providers. For example, some people in Maryland who became Medicaid-eligible upon expansion might be more willing to rely on primary care physicians or non-emergency clinics, as opposed to the ED, for most of their healthcare needs. However, similar individuals in Illinois might react in the opposite way. So, assuming people who live relatively close to each other are more likely to share similar reactions to environmental or policy changes, we chose states that are relatively close to Maryland and implemented Medicaid expansion on the same day.

As listed in Table A1, eleven states and one district are geographically close to Maryland. However, Connecticut (CT), District of Columbia (DC), New Jersey (NJ), Pennsylvania (PA), and Virginia (VA) participated in Medicaid expansion but not with a start date of January 1, 2014. North Carolina (NC) did not implement Medicaid expansion. After ruling out those states and one district, we had five states to consider: Delaware (DE), Massachusetts (MA), Rhode Island (RI), Vermont (VT), and West Virginia (WV). Massachusetts implemented Medicaid expansion on the same day as Maryland did; however, it may have subsequently experienced a policy change. In January 2015, one year after the implementation of Medicaid expansion, the newly elected governor’s primary focus was to remove from the Medicaid rolls those who were automatically enrolled in MassHealth in 2014.^9^ Since this happened during our study period, we decided not to include Massachusetts in the control group. Vermont did participate in Medicaid expansion on January 1, 2014. However, throughout 2014 and 2015, there were technical problems with its state exchange that made it impossible to identify an individual’s eligibility for Medicaid coverage while renewing enrollment.^10^ Through a waiver from the federal government, the state changed its policy and allowed individuals to renew their enrollment automatically until 2016, when the state fixed its technical problems. Therefore, three geographically proximate states, West Virginia, Delaware, and Rhode Island, were chosen for comparison as the control group. We believe our control group can adjust for the impact of Medicaid expansion on Maryland EDs.

**A3. ED MEASURES FROM THE CENTERS FOR MEDICARE & MEDICAID SERVICES**

Table A3 lists multiple ED throughput efficiency measures defined by the Centers for Medicare & Medicaid Services (CMS). From these measures, we choose ED1b as the focus of our study. ED1b is the median time (in minutes) that inpatients (those who are subsequently admitted to the facility) spend from the time of arrival in the ED to the time of departure from the ED into the hospital or other healthcare facility.

**Table A3.^11^** Timely and Effective Care – Emergency Department Throughput Measures.

| Measure ID | Technical Measure Title |
| --- | --- |
| EDV | ED volume |
| ED1b | Median time from ED arrival to time of departure from the ED for patients admitted to the hospital |
| ED2b | Median time from admit decision to time of departure from the ED for patients admitted to inpatient status |
| OP18b | Median time from ED arrival to ED departure for discharged patients |
| OP20 | Door to diagnostic evaluation by a qualified medical professional |
| OP21 | Median time to pain management for long bone fracture |
| OP22 | Patient left without being seen |
| OP23 | Head CT scan results for acute ischemic stroke or hemorrhagic stroke who received head CT scan interpretation within 45 minutes of arrival |

In general, inpatients who are admitted to a hospital through the ED have serious or life-threatening ailments and require immediate attention, hospital admission, or surgery. Compared with wait time (CMS measure ED2b), total time spent in the ED is a crucial measure, since reducing this time can potentially improve both medical treatment quality and patient access to treatment whereas prolonging this time can cause problems such as overcrowding. ED overcrowding may lead to more problems, such as ambulance refusals, unpleasant communications and interactions, and limited ability to respond to other emergencies.^12^ Another reason we consider total time spent in the ED over wait time is that after the first quarter of 2018, CMS no longer collected data for outpatient wait time in the ED. Since CMS only has reports of the total time spent by outpatients in EDs in Maryland for the years after 2014 (OP18b, Table A3), we cannot study outpatient measures. Therefore, we use ED1b as our study measure.

**A4. MODEL AND SETUP**

We formatted the dataset into an unbalanced panel dataset and implemented the mixed effects model with a state-fixed effect, a hospital-level random effect, and state-level heterogeneity.

**Formula A.1:** Complete Model.

*ED1b_it_*=*const*+(*β*×*ri_i_*)+(*δ*×*wv_i_*)+(*𝜙*×*de_i_*)+(*φ*×*bed_it_*)+(*γ*×*t_it_*)+(*ρ*×*tt_it_*)+(*𝜆*×*medicaid_ist_*)+(*𝜇*×*edvperpop_ist_*)+ *ε_it_*

where *tt_it_*=*GB*×*t_it_*×*period_it_*.

The subscript *s* indicates the data are state-level while *t* indicates that the variable changes over time periods and *i* is the hospital index.

The dependent variable *ED1b* is the median time a person spends in the ED from arrival at the ED to the time of admission as an inpatient to the hospital or other healthcare facility.

The indicator variables, *ri*, *wv*, and *de,* represent Rhode Island, West Virginia, and Delaware respectively. *GB* is an indicator variable that indicates if the hospital is under GBR. It is 0 for all hospitals in the control group and 1 for hospitals in the treatment group.

The variable *bed* is the number of beds in the hospital. This number varies over time during the study. Since the CMS Hospital Cost report is not on a fiscal year cycle like the CMS Hospital Compare reports, we choose the data that gave us the largest overlapping time period. For example, if a hospital’s ED1b score is from March 2012 to March 2013, then, since the report date varies across hospitals, we take the *bed* value of this hospital from the CMS Hospital Cost report for fiscal year 2012.

Time is captured by *t*, a variable with four possible values (-2, -1, 1, 2) as described in Table A4. January 1, 2014 is the 0 point, so when *t* equals 1, the ED1b score was measured during the first year after GBR implementation; when *t* equals -2, the ED1b score was measured during the two years prior to January 1, 2014. The variable *period* is a dummy variable that equals 1 for the post-treatment period and 0 otherwise. The variable *tt* is an interaction term between time, treatment, and post-treatment period dummy variables. It is the key variable describing the effect of GBR on the ED1b performance of hospitals in Maryland.

The variable *medicaid* is the Medicaid enrollment percentage of the population in the four states from 2012 to 2015. Medicaid expansion could affect ED usage, and we intend to control the impact of change in Medicaid enrollment on ED1b performance. Since the Kaiser Family Foundation (KFF) only provides the total number of individuals enrolled, we divide total enrolled in Medicaid in a given year by the total state population in that same year.^13^ KFF does not have a Medicaid enrollment report for 2012. To estimate this missing data, we calculate the Medicaid enrollment rates for 2011 and 2013 and use the average of the two rates for the missing 2012 data.

**Table A4.** Definitions of Variables *t* and *tt.*

| Measurement period | *t*(Time) value | *tt* value for treatment group |
| --- | --- | --- |
| 1/1/2012-9/30/2012 | -2 | 0 |
| 10/1/2012-9/30/2013 | -1 | 0 |
| 4/1/2014-3/31/2015 | 1 | 1 |
| 4/1/2015-3/31/2016 | 2 | 2 |

To capture ED utilization per thousand residents, we use the variable *edvperpop*, annual Hospital ED Visits per Thousand Population.^1,14^ The ideal variable would be historical hospital-level data on ED utilization. Because we do not have access to those data, we use *edvperpop*.

The error terms *ε_it_*=*η_i_*+*υ_it_* have two parts. The variable *η_i_* is the random effect that has a normal distribution *N* (0, *σ_η_^2^*) and satisfies the random effects assumption. The random effect is modeled to account for the deviations for each individual hospital from the state-level fixed effect. The variable *υ_it_* is a random error term that is *N* (0, *σ_υ_^2^*). We assume heterogeneity across states and that error terms share the same normal distribution within states.

**A5. MODEL ANALYSIS DETAILS**

A paper published in 2015 studied the utilization of the statewide Emergency Medical Services (EMS) protocols.^15^ The authors found wide variation in the format and characteristics of these protocols and the recognition of specialty receiving centers for patients with time-sensitive illnesses. In the United States, many EMS protocols are listed on the public website *EMS Protocols*, which arranges the protocols into categories based on states or counties.^16^ We believe that this variation in EMS protocols may be partly responsible for the differences in ED1b scores that we observed across the four states in our study. It is also possible that different hospitals within the same state might respond to EMS protocols differently. Therefore, we decided to adopt a model mixing state-level fixed effects and hospital-level random effects. To test the fitness and correctness of the mixed effects model, we implemented the following procedures.

First, we tested the presence of the fixed effects and random effects on the following model:

**Formula A.2:** First Layer Fixed Effects and Random Effects Test Model.

*ED1b_it_*=*const*+(*φ*×*bed_it_*)+(*γ*×*t_it_*)+(*ρ*×*tt_it_*)+(*𝜆*×*medicaid_ist_*)+(*𝜇*×*edvperpop_ist_*)+ *ε_it_*

where the error terms *ε_it_* follow the same normal distribution.

Table A5 shows the results of the general fixed effects test. The value of the *F*-test is 18.02 and the critical value at the 0.05 significance level is 1.31, which implies that fixed effects exist. As previously discussed, we believe the ED1b score varies across states partially because of the variation in EMS protocols. However, from a data perspective, we double-checked the fitness of the fixed effects over random effects model.

Table A6 shows the regression results of the general random effects model. Its Breusch and Pagan LM test value is 326.64, implying that random effects model should be considered. We then employed the Hausman test on the two regression results. The null hypothesis is that the difference in coefficients is not systematic. The Chi-square value is 16.54 and the critical value at the 0.05 significant level is 11.07, which prompted our choice of fixed effects over random effects.

Instead of choosing hospital-level fixed effects, we used state-level fixed effects for two reasons. First, our study involves state EMS protocols and, as discussed above, studies have shown wide variation in these protocols across states. It is reasonable to set state-level fixed effects to capture the difference in ED1b scores caused by EMS variation. Second, our study contains a total of 353 observations. If we used hospital-level fixed effects, we would need to add 97 fixed effects variables, one for each hospital. The ratio of available observations and number of variables would be too small and violate the requirement of the number of subjects per variable in linear regression analysis, thus making state-level fixed effects the better option.

After capturing the state-level fixed effects, we explored into the variation in the ED1b score of each hospital from its state-level fixed effects. At the hospital level, the difference in responses to state EMS protocols could be caused by many features, such as location, hospital service area population, administration, and layout of hospital buildings. We believe a random effects model should be able to capture this variation. To verify this, we used the following model:

**Formula A.3:** Second Layer Fixed Effects and Random Effects Test Model.

*ED1b_it_*=*const*+(*β*×*ri_i_*)+(*δ*×*wv_i_*)+(*𝜙*×*de_i_*)+(*φ*×*bed_it_*)+(*γ*×*t_it_*)+(*ρ*×*tt_it_*)+(*𝜆*×*medicaid_ist_*)+(*𝜇*×*edvperpop_ist_*)+ *ε_it_*

where the error terms *ε_it_* follow the same normal distribution.

Table A7 shows that, after introducing the state-level fixed effects, we could use another layer of fixed effects to represent the ED1b score of each hospital from its state-level fixed effects. Table A8 shows the test of random effects on the regression model after introducing the state-level fixed effects. The Breusch and Pagan LM test value is 331.12, which implies another layer of random effects should be considered. Then we ran the Hausman test on the two regression results. The null hypothesis is that there is no systematic difference in coefficients. The Hausman test value is 1.68 and the critical value at the 0.05 significance level is 11.07, so we failed to reject the null hypothesis. Therefore, we decided to use hospital-level random effects to capture the variation in the ED1b score of each hospital from its state-level fixed effects.

Another point we considered was heterogeneity. In our study, there are 353 observations. We regrouped the dataset by states and calculated the standard errors of the ED1b scores. The overall estimated standard error of the ED1b scores is 84.5 for Maryland and 59.2, 62.5, and 71.4 for West Virginia, Delaware, and Rhode Island, respectively. These differences imply heterogeneity across states.

The results of our mixed effects model with state-level heterogeneity are presented in Table 4 in the paper.

**Table A5.** Regression Results with Hospital-Level Fixed Effects.

| Variables | Coefficient | Confidence Interval (95%) |
| --- | --- | --- |
| *t* | 1.317345 | (-1.851913, 4.486603) |
| *tt* | 10.30092 ** | (2.987586, 17.61425) |
| *bed* | 0.3378979 ** | (0.1271581, 0.5486378) |
| *medicaid* | -0.5592615 | (-2.14773, 1.029206) |
| *edvperpop* | -0.0098834 | (-0.2239198, 0.204153) |
| *const* | 266.0747 *** | (140.3394, 391.8099) |

Fixed Effects *F*-Test: *F*-value=18.02, (Critical Value =1.31)

*** *p*-value≤0.001, ** *p*-value≤0.01

**Table A6.** Regression Results with Hospital-Level Random Effects.

| Variables | Coefficient | Confidence Interval (95%) |
| --- | --- | --- |
| *t* | -0.5344875 | (-3.543396, 2.474421) |
| *tt* | 9.2035 * | (1.968284, 16.43872) |
| *bed* | 0.2387744 *** | (0.1722657, 0.3052831) |
| *medicaid* | -0.3154984 | (-1.852904, 1.221907) |
| *edvperpop* | -0.3510833 *** | (-0.4709234, -0.2312431) |
| *const* | 462.1924 *** | (382.2349, 542.15) |

Breusch and Pagan LM test: 326.64.

*** *p*-value≤0.001, ** *p*-value≤0.01

**Table A7.** Regression Results with State-Level Fixed Effects and Hospital-Level Fixed Effects.

| Variables | Coefficient | Confidence Interval (95%) |
| --- | --- | --- |
| *ri* | omitted |  |
| *wv* | omitted |  |
| *de* | omitted |  |
| *t* | 1.317345 | (-1.851913, 4.486603) |
| *tt* | 10.30092 ** | (2.987586, 17.61425) |
| *bed* | 0.3378979 ** | (0.1271581, 0.5486378) |
| *medicaid* | -0.5592615 | (-2.14773, 1.029206) |
| *edvperpop* | -0.0098834 | (-0.2239198, 0.204153) |
| *const* | 266.0747 *** | (140.3394, 391.8099) |

Fixed Effects F-Test: *F*-value=18.02, (Critical Value =1.31)

*** *p*-value≤0.001, ** *p*-value≤0.01

**Table A8.** Regression Results with State-Level Fixed Effects and Hospital-Level Random Effects.

| Variables | Coefficient | Confidence Interval (95%) |
| --- | --- | --- |
| *ri* | -54.47875 * | (-100.6191, -8.338395) |
| *wv* | -106.9579 *** | (-162.1082, -51.80748) |
| *de* | 6.510064 | (-48.07497, 61.0951) |
| *t* | 1.393201 | (-1.73705, 4.523453) |
| *tt* | 9.466872 ** | (2.3587, 16.57504) |
| *bed* | 0.2096206 *** | (0.1431307, 0.2761104) |
| *medicaid* | -0.6514587 | (-2.221543, 0.9186259) |
| *edvperpop* | -0.0064578 | (-0.2185943, 0.2056787) |
| *const* | 342.5963 *** | (242.8828, 442.3098) |

Breusch and Pagan LM test: 331.12.

*** *p*-value≤0.001, ** *p*-value≤0.01, **p*-value≤0.05

**A6. SENSITIVITY ANALYSES**

We conducted four types of sensitivity analysis on the original mixed effects model. The first analysis assessed whether our treatment effects estimates were sensitive to the length of the first report period. In our study, we used four CMS Hospital Compare data reports. The first report covers the 9-month period from January 1, 2012 to September 30, 2012, and the remaining three cover 12-month periods. We introduced a new variable *length* (in months) to assess the impact of report period length. As shown in Table A9, the regression estimator for length (*length*=-1.341053, *p*-value=0.384) implies that the shorter time period of the first report does not impact the ED1b scores which can be excluded from the model.

Second, to consider whether our estimates were affected by the change in available healthcare resources caused by incidents such as hospital closures or shifts in access to care, we introduced two state-level variables: *nurseperpop* as the total number of registered nurses per thousand population 2012-2015^1,17^ and *bedperpop* as the hospital beds per thousand population 2012-2015^1,18,19^. To generate these two variables, we divided total number of registered nurses and hospital beds by the total state population in the same year. Table A10 summarizes the regression results with these two variables.

Third, to assess whether our estimates were sensitive to each state in the control group, we conducted three sensitivity analysis using three alternative control groups. In the first alternative control group, we removed the hospitals in West Virginia counties with populations less than 45,000 which is the population of the smallest MD county in our study. This leaves 18 WV hospitals in the control group. In the other two alternative control groups, we removed all hospitals from RI and DE, respectively. Table A11 summarizes the regression results using three alternative control groups. Table A12 summarizes all sensitivity analysis estimates. The regression results show that using the three alternative control groups is consistent with using all three states’ hospitals or with including the length of report periods.

Fourth, we conducted a robustness check on the relationship between the number of hospital beds and the ED1b score. In the main paper (Figure 1), we concluded that the larger the hospital the longer it takes for a patient to leave the ED. Realizing that the hospitals with more than 500 beds might have an impact on the conclusion, we decided to remove these large hospitals and rerun the experiments. The large hospitals are Christiana Care Health Services, Inc. (DE), The Johns Hopkins Hospital (MD), Charleston Area Medical Center (WV), University of Maryland Medical Center (MD), and Rhode Island Hospital (RI). Table A13 shows the coefficient estimates after removing the large hospitals. The number of hospital beds (coef. = 0.270943, *p*-value<0.001) is still positively associated with a hospital’s ED1b score. Figure A2 is the scatter plot for ED1b score versus total number of hospital beds. This robustness check supports the conclusion the larger the hospital, the longer it takes in the ED.


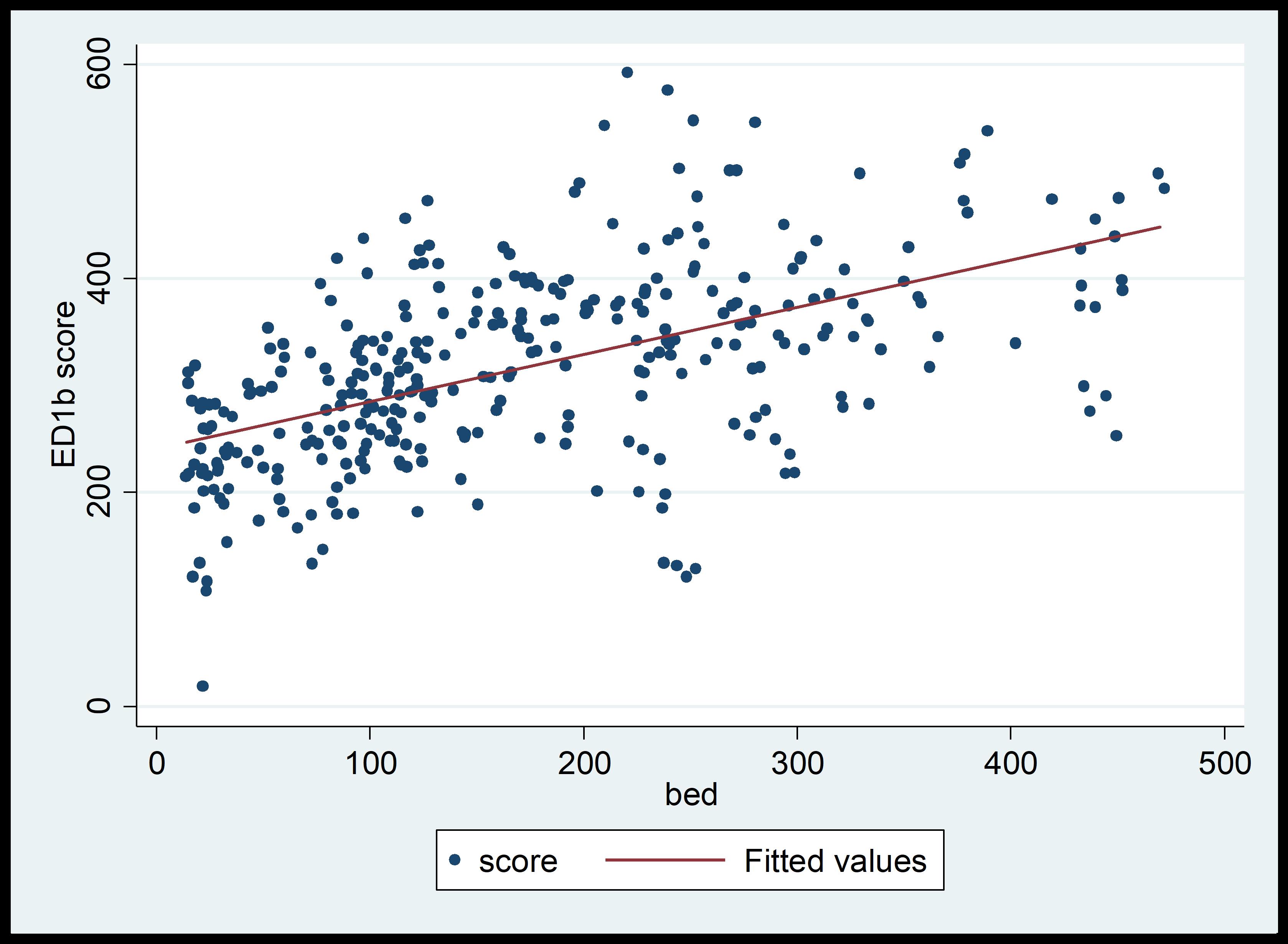
**Figure A2.** Scatter Plot of ED1b Scores vs. Total Number of Hospital Beds (Large Hospitals Removed).

**Table A9.** Mixed Model vs. Mixed Model with the Length of the First Report.

| Variables | Coefficient (without *length*) | Coefficient (with *length*) |
| --- | --- | --- |
| *ri* | -54.47875 *** | -49.56627 *** |
| *wv* | -106.9579 ** | -96.21136 *** |
| *de* | 6.510064 | 9.848581 ** |
| *t* | 1.393201 *** | 2.277189 ** |
| *tt* | 9.466872 *** | 8.480697 *** |
| *bed* | 0.2096206 *** | 0.2099377 *** |
| *medicaid* | -0.6514587 | -0.7847283 |
| *edvperpop* | -0.0064578 | -0.060419 |
| *const* | 342.5963 *** | 382.9416*** |
| *length* |  | -1.341053 |

*** *p*-value≤0.001, ** *p*-value≤0.01

**Table A10.** Mixed Model vs. Mixed Model with the Total Number of Registered Nurses per Thousand Population and the Hospital Beds per Thousand Population.

| Variables | Coefficient (without new variables) | Coefficient (with two new variables) |
| --- | --- | --- |
| *ri* | -54.47875 *** | -5.973945 |
| *wv* | -106.9579 ** | -68.51933 |
| *de* | 6.510064 | 51.51461*** |
| *t* | 1.393201 *** | 6.173958*** |
| *tt* | 9.466872 *** | 8.825362*** |
| *bed* | 0.2096206 *** | 0.208171*** |
| *medicaid* | -0.6514587 | -1.5871*** |
| *edvperpop* | -0.0064578 | 0.094159 |
| *nurseperpop* |  | -16.68766*** |
| *bedperpop* |  | -18.08304 |
| *const* | 342.5963 *** | 487.0217*** |

*** *p*-value≤0.001, ** *p*-value≤0.01

**Table A11.** Alternative Control Groups.

| Variables | Coefficient (Mixed effects model) | Coefficient (partial WV) | Coefficient (drop RI) | Coefficient (drop DE) |
| --- | --- | --- | --- | --- |
| *ri* | -54.47875 *** | -50.90472 *** | omitted | -53.62928 *** |
| *wv* | -106.9579 ** | -83.30162 ** | -118.6393 ** | -104.8781 ** |
| *de* | 6.510064 | 10.20236 | -35.05085 *** | omitted |
| *t* | 1.393201 *** | 0.0458752 | -2.175164 *** | 1.340226 *** |
| *tt* | 9.466872 *** | 10.90678 *** | 8.422524 *** | 9.85151 *** |
| *bed* | 0.2096206 *** | 0.1931021 * | 0.219232 *** | 0.2417658 *** |
| *medicaid* | -0.6514587 | -0.4393541 | 5.468769 *** | -0.6160108 |
| *edvperpop* | -0.0064578 | -0.0507006 | 0.1990481 ** | 0.0057883 |
| *const* | 342.5963 *** | 360.979 *** | 158.3014 *** | 328.1214 *** |

*** *p*-value≤0.001, ** *p*-value≤0.01, **p*-value ≤0.05

**Table A12**. Summary of Sensitivity Analysis Estimates.

| Model | GBR Effect Estimate | 95% Confidence Interval |
| --- | --- | --- |
| Mixed Model | 9.466872 *** | (7.062948, 11.8708) |
| Adding Length of Report Period | 8.480697 *** | (7.851343, 9.110051) |
| Adding Registered Nurses per Thousand Population and Hospital Beds per Thousand Population | 8.825362 *** | (7.197024, 10.4537) |
| Control Group with Partial WV | 10.90678 *** | (6.785388, 15.02817) |
| Control Group without DE | 9.85151 *** | (7.934328, 11.76869) |
| Control Group without RI | 8.422524 *** | (7.44502, 9.400028) |

*** *p*-value≤0.001

**Table 13.** Coefficient Estimates After Removing Large Hospitals.

| Variables | Coefficient | Coefficient (removing large hospitals) |
| --- | --- | --- |
| *ri* | -54.47875 *** | -42.35795 * |
| *wv* | -106.9579 ** | -86.42894 * |
| *de* | 6.510064 | 38.81425 ** |
| *t* | 1.393201 *** | 1.13967 ** |
| *tt* | 9.466872 *** | 10.49142 *** |
| *bed* | 0.2096206 *** | 0.270943 *** |
| *medicaid* | -0.6514587 | -0.6792324 |
| *edvperpop* | -0.0064578 | -0.0400381 |
| *const* | 342.5963 *** | 338.1285 *** |

*** *p*-value≤0.001, ** *p*-value≤0.01, **p*-value ≤0.05

**A7. REFERENCES**

1. US Census Bureau https://www.census.gov/. Accessed March 1, 2018.
2. CMS. <https://www.cms.gov/Research-Statistics-Data-and-Systems/Downloadable-Public-Use-Files/Cost-Reports/Hospital-2010-form.html>. Accessed March 1, 2018.
3. Giuriceo K, Haber S, Beil H, et al. *Evaluation of the Maryland All-Payer Model Second Annual Report*. August 2017. CMS Contract Number HHSM-500-2010-00021I, Task Order HHSM-500-T0013.
4. KFF. <https://www.kff.org/health-reform/issue-brief/states-getting-a-jump-start-on-health/>. Accessed March 1, 2018.
5. KFF. https://www.kff.org/health-reform/state-indicator/state-activity-around-expanding-medicaid-under-the-affordable-care-act/. Accessed November 30, 2018.
6. KFF. https://www.kff.org/medicaid/issue-brief/the-effects-of-medicaid-expansion-under-the-aca-updated-findings-from-a-literature-review-march-2018/. Accessed October 1, 2018.
7. Klein EY, Levin S, Toerper MF, et al. The effect of Medicaid expansion on utilization in Maryland emergency departments. *Ann Emerg Med.* 2017;70(5):607-14.
8. Sharma AI, Dresden SM, Powell ES, et al. Emergency department visits and hospitalizations for the uninsured in Illinois before and after Affordable Care Act insurance expansion. *J Community Health*. 2017;42(3):591-7.
9. HealthInsurance.org <https://www.healthinsurance.org/massachusetts-medicaid/>. Accessed October 1, 2018.
10. HealthInsurance.org <https://www.healthinsurance.org/vermont-medicaid/>. Accessed October 1, 2018.
11. CMS. Title: 15-2. <https://www.cms.gov/Regulations-and-Guidance/Guidance/Manuals/Paper-Based-Manuals-Items/CMS021935.html>. Accessed March 1, 2018.
12. Specifications Manual for National Hospital Inpatient Quality Measures. Version 5.3. The Quality Net page. http://www.qualitynet.org/dcs/ContentServer?c=Page&pagename=QnetPublic%2FPage%2FQnetTier1&cid=1121785350606. Accessed March 1, 2018.
13. CMS <https://data.medicare.gov/data/hospital-compare>. Accessed March 1, 2018.
14. KFF <https://www.kff.org/other/state-indicator/emergency-room-visits-by-ownership/>. Accessed March 1, 2018.
15. Kupas DF, Schenk E, Sholl JM, et al. Characteristics of statewide protocols for emergency medical services in the United States. *Prehosp Emerg Care.* 2015 Apr-Jun; 19(2):292-301.
16. EMS Protocols. http://www.emsprotocols.org/. Accessed March 1, 2018.
17. Bureau of Labor Statistics. <https://www.bls.gov/oes/current/oes291141.htm>. Accessed March 1, 2018.
18. 1999 - 2015 AHA Annual Survey. Health Forum, LLC, an affiliate of the American Hospital Association. Special data request, 2016.
19. KFF <https://www.kff.org/other/state-indicator/beds-by-ownership>. Accessed March 1, 2018.
